# Supplementary material for: Amylin-Calcitonin receptor signaling in the medial preoptic area mediates affiliative social behaviors in female mice
Source: Nat Commun. 2022 Feb 8;13:709. doi: 10.1038/s41467-022-28131-z (PMC8825811; doi:10.1038/s41467-022-28131-z)
Supplement: Supplementary file 1 — Supplementary information [file 41467_2022_28131_MOESM1_ESM.pdf]

Supplementary information for

Amylin-Calcitonin receptor signaling in the medial preoptic area mediates affiliative social behaviors in female mice

Kansai Fukumitsu*, Misato Kaneko, Teppo Maruyama, Chihiro Yoshihara, Arthur J. Huang, Thomas J. McHugh, Shigeyoshi Itohara, Minoru Tanaka, and Kumi O Kuroda*

***Corresponding author:** Kansai Fukumitsu and Kumi O. Kuroda
E-mail: kansai.fukumitsu@riken.jp, kumi.kuroda@a.riken.jp

This PDF file includes:

Figs. S1 to S8
Supplementary Discussions

Supplementary Figures

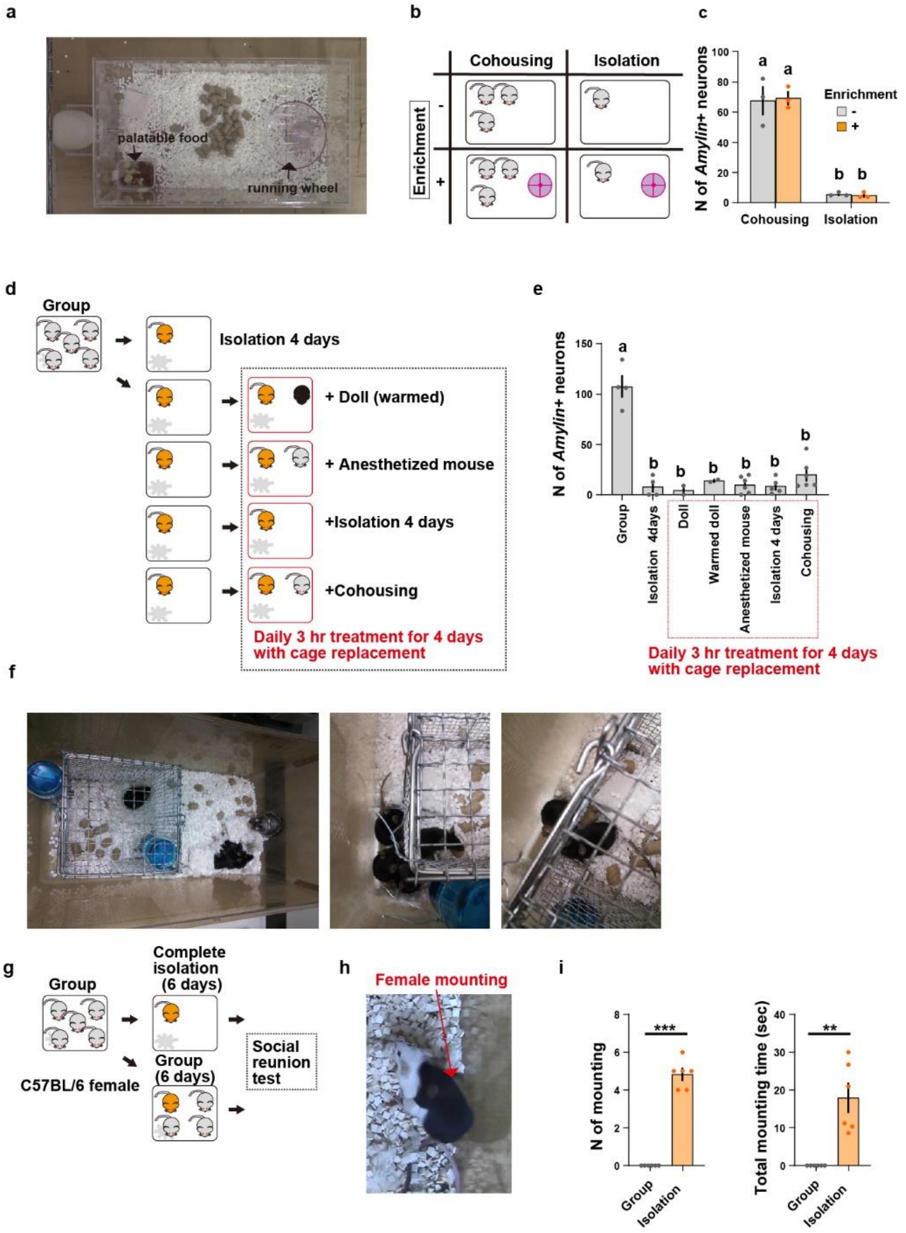

Supplementary Figure 1 Additional experiments performed in C57BL/6 female mice. (**a–c**) Environmental enrichment does not affect the expression of *Amylin* mRNA in the MPOA. (**a**) The environmentally-enriched cage included a running wheel and palatable food that was replaced every day. (**b**) Experimental procedure. Before histochemical analyses, female mice were cohoused or single-housed with or without environmental enrichment for 6 days. (**c**) Quantification of the number of *Amylin* mRNA-expressing neurons in the MPOA (n = 3 mice per group). The letters indicate significant differences (two-way ANOVA with Sidak's multiple comparison test, $p < 0.001$). (**d–e**) Distal sensory stimulations do not maintain *Amylin* expression. (**d**) An anesthetized cage mate, a cold or

a warmed-up soft felt mock mouse was introduced daily in the subject's cage for 3 h during 4 consecutive days. (**e**) The number of *Amylin* mRNA-expressing neurons was counted (group, isolation 4 days: $n = 4$ mice, isolation 4 days and cage replacement: $n = 5$, anesthetized cage mates, cohousing 3 h/day: $n = 6$, cold or warmed-up mock mouse: $n = 2$). The letters indicate significant differences (one-way ANOVA with Tukey's multiple comparison test, $p < 0.001$). (**f**) Additional data for Fig. 1p. A group of C57BL/6 female mice was habituated for 6 days to a large test cage containing a wire-mesh compartment. Then, one female was segregated in the compartment. Soon after containment, the isolated female exhibited behaviors such as vigorously biting, digging beneath, and climbing up the compartment walls. The isolated female then became inactive (for the time course, see below descriptions as well as Fig. 2e and S2), and slept in contact with the mesh wall and the cage mates huddled near the compartment walls. (**g–i**) Female mounting behavior exhibited in the cohousing test by 6-days isolated and group-housed mice. (**h**) Representative picture of the mounting behavior of a socially isolated and group-housed C57BL/6 female mouse. (**i**) Number (N) of female mountings and total mounting time during a 30-min period ($n = 6$ mice in each group). Asterisks indicate significant differences between two groups (Welch's unpaired *t*-test, $^{**}p < 0.01$, $^{***}p < 0.001$). Graphs show mean ± SEM. See Supplementary Data for exact p values and details of statistical analyses.

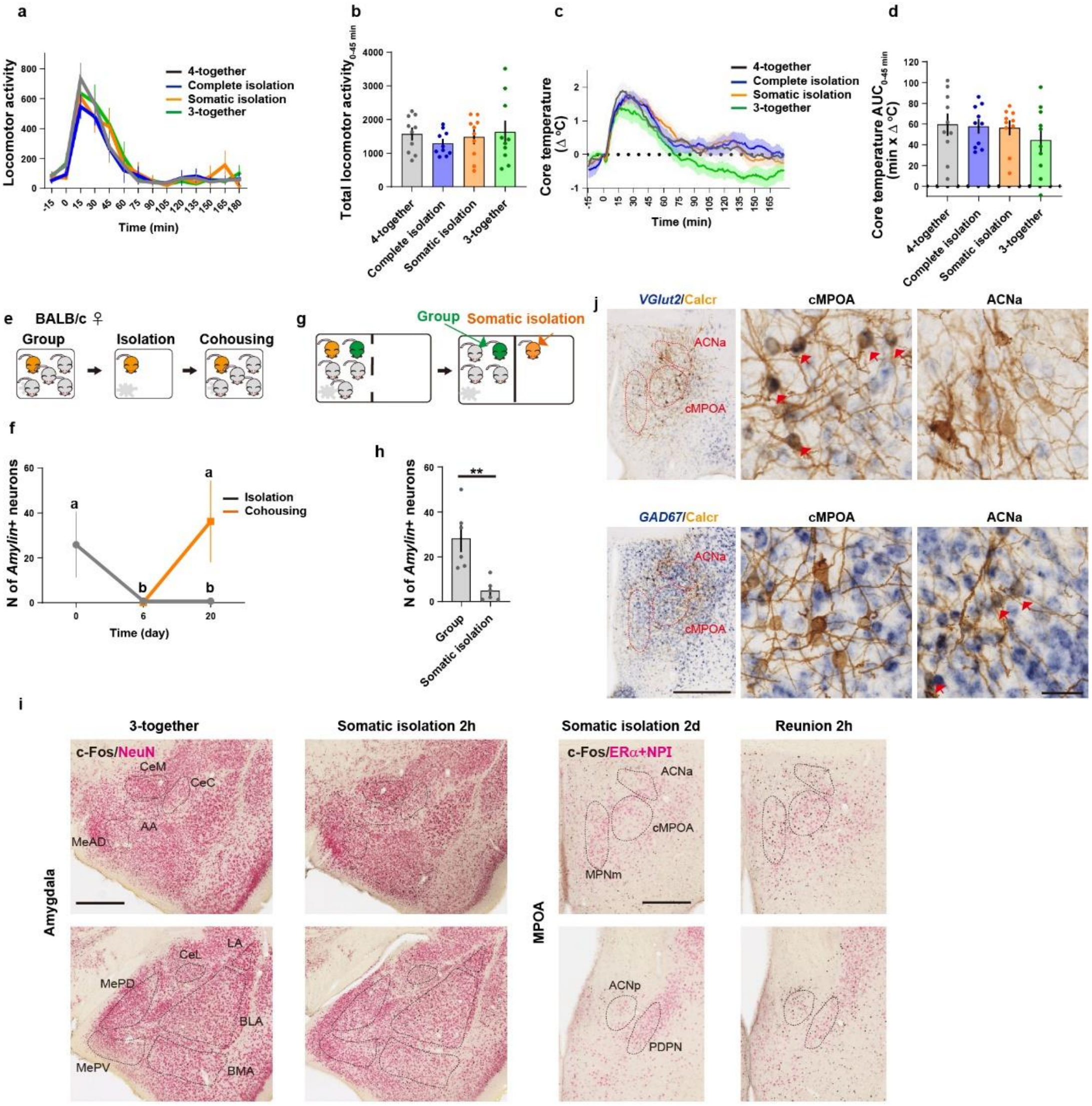

Supplementary Figure 2 Supportive experiments for Fig. 1, Fig. 2 and Fig. 4 in BALB/c female mice.

(**a–b**) Additional data for Fig 2. Time course analysis of the locomotor activity (**a**) and total locomotor activity displayed over 45 min (**b**). (**c–d**) Changes in the core temperature induced by social isolation (**c**) and the corresponding area under curve for a 45-min period (**d**). For each group: n = 10 mice. Time bins: 1 min. There was no significant difference between groups (one-way ANOVA). (**e–f**) Additional data for Fig. 1e. Group-housed BALB/c female mice were isolated for 6 days, and then cohoused for 2 weeks. *Amylin* mRNA levels were decreased by social isolation and increased by cohousing. (**f**) Sections were stained by ISH using *Amylin* probe. Amylin+ neurons were quantified in the MPOA (n = 6 mice per group). The letters indicate significant differences (Kruskal-Wallis with Dunn's multiple comparison test, $p < 0.05$). (**g–h**) Six days of in-cage isolation completely

abolished *Amylin* mRNA-expression in the MPOA, in a similar fashion than single-housing did (Fig. 1q). (**h**) Sections were stained by ISH using *Amylin* probe. Amylin+ neurons were quantified in the MPOA (n = 6 mice per group). Asterisks indicate significant differences between two groups (Mann-Whitney U test, two-sided, $^{**}p < 0.01$). (**i**) Representative images for Fig. 4. Coronal sections were stained for c-Fos (black), and NPI and ERα (both in red) in the MPOA or NeuN (red) in the amygdala. Scale bars, 500 μm. (**j**) Co-expression of Calcr and VGLUT2 or GAD67 in distinct preoptic subregions. *VGlut2* or *Gad67* mRNAs (blue) were stained by ISH and Calcr (brown) was detected with specific antibodies. Arrowheads indicate double-labeled cells. Scale bars, 500 μm (left) and 50 μm (middle and right). Graphs show mean ± SEM. See Supplementary Data for exact p values and details of statistical analyses.

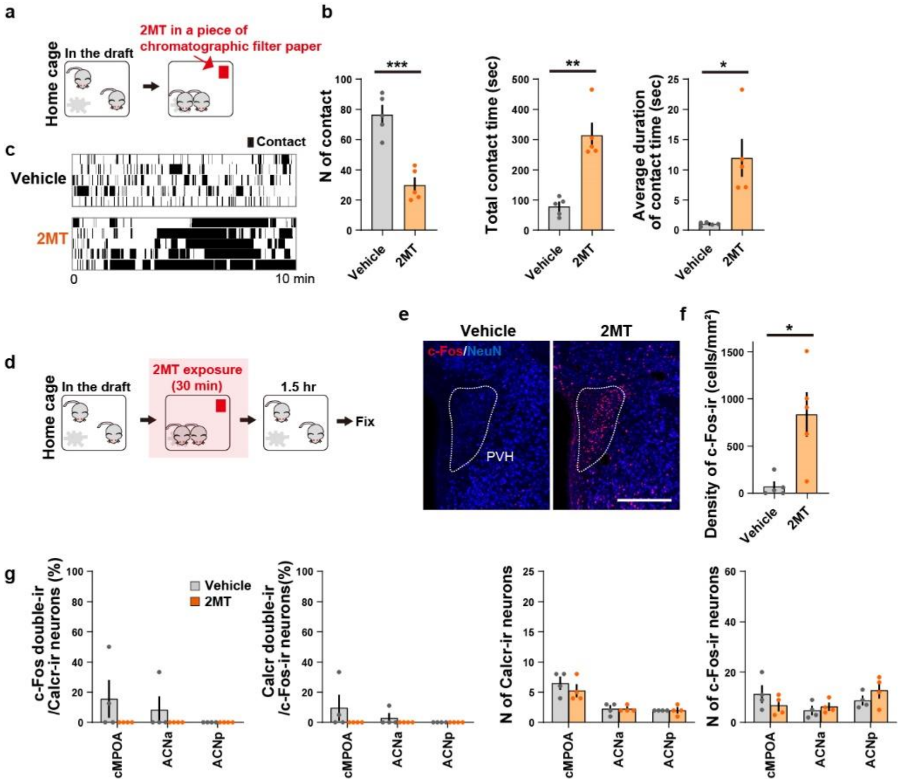

Supplementary Figure 3 Predator odor induces defensive huddle.

(**a**) Schematics of the defensive huddle test using a stressful predator odor condition. Two female mice were exposed in the home cage to a piece of chromatographic filter paper soaked with 2MT or vehicle. (**b**) Quantification of the number of contacts, total contact time, and average duration of contact during 10 min (*n* = 5 mice per group). (**c)** Raster plots of social contact bouts from mice treated with vehicle or 2MT. (**d**) Schematic of the experimental procedure. (**e**) Coronal sections were stained for c-Fos (red) and NeuN (blue). Scale bar, 250 µm. (**f**) Density of c-Fos-immunoreactive (c-Fos-ir) neurons in the PVH after 2MT treatment (*n* = 5 mice per group). (**g**) Percentage of Calcr-ir neurons expressing c-Fos, percentage of c-Fos-ir neurons expressing Calcr, number of Calcr-ir or c-Fos-ir neurons in each subregion after 2MT treatment (*n* = 4 mice per group). Asterisks in **(b)** and **(f)** indicate significant differences between two groups (Welch's unpaired t-test, *$p < 0.05$, **$p < 0.01$, ***$p < 0.001$). Graphs show mean ± SEM. See Supplementary Data for exact *p* values and details of statistical analyses.

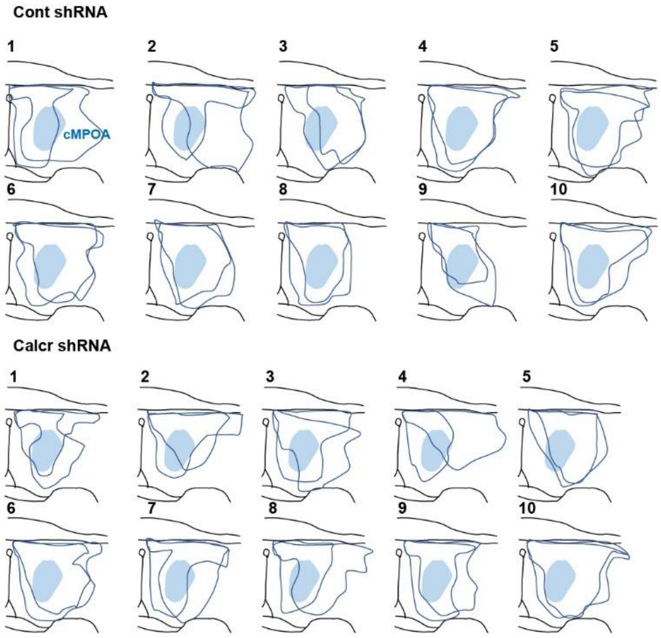

Supplementary Figure 4 Supportive data for Fig. 6. Knockdown of Calcr in the cMPOA target areas.

Schematic coronal sections showing the cMPOA (blue) and the infection site of AAV5-*hH1-Calcr shRNA* or *scrambled shRNA-CAG-EGFP* in the MPOA.

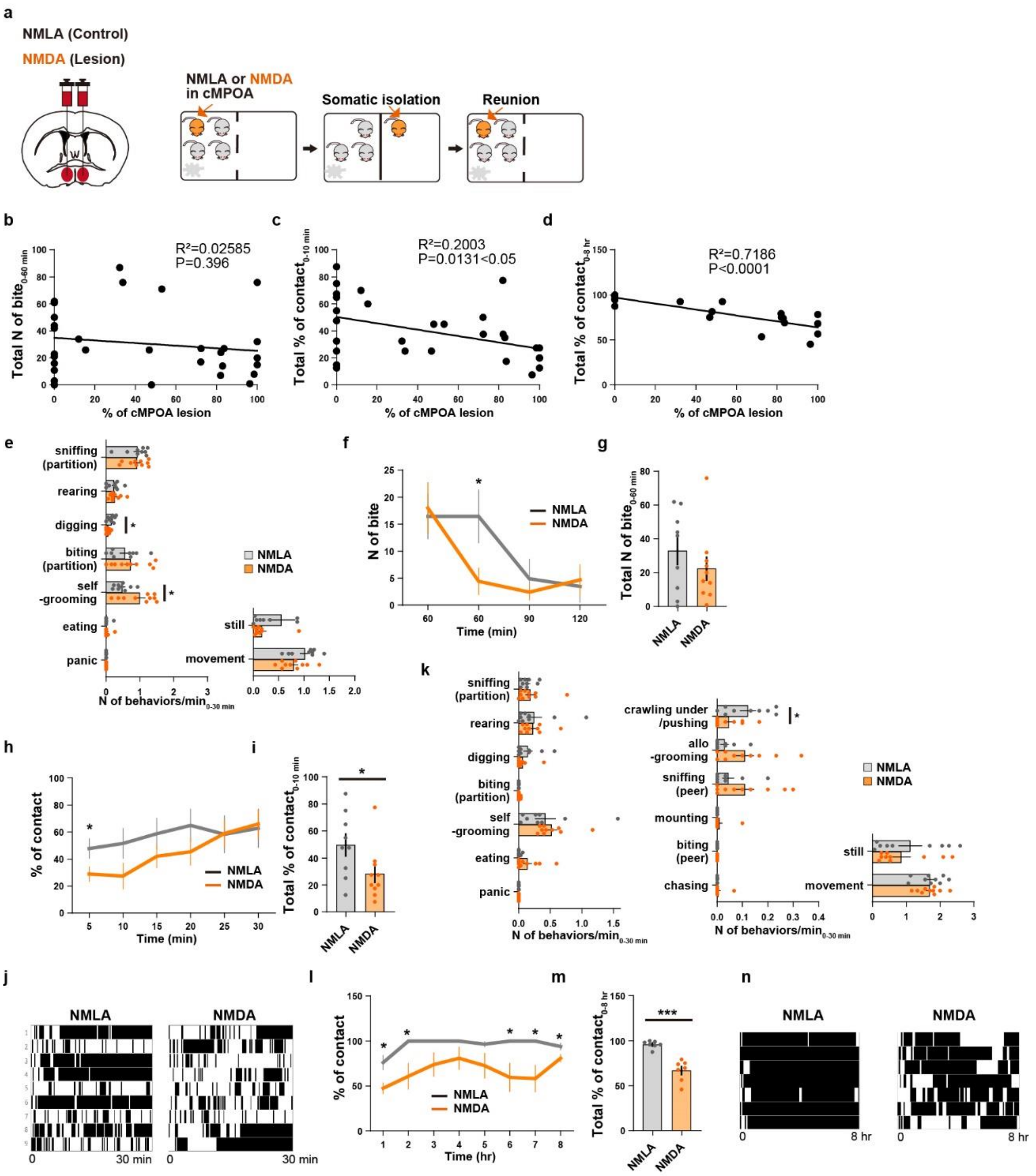

Supplementary Figure 5 The cMPOA facilitates social contact behaviors

(**a**) Procedure of excitotoxin lesion experiments. N-methyl-D-aspartic acid (NMDA) or N-methyl-L-aspartic acid (NMLA) (control) was bilaterally injected into the MPOA. The somatic isolation test was performed 1 week later. Two days after, the social reunion test was carried out. (**b–d**) Correlation between behaviors and the extent of the cMPOA lesion. Social contact was negatively corelated with cMPOA lesion. Pearson correlation test, two-sided (b, c: n = 30 mice, d: n = 22 mice). (**e–n**) Successfully lesioned samples (more

than 80 % of the cMPOA destroyed) were analyzed. (**e**) Quantification (number of occurrences, N, per min) of behaviors seen under somatic isolation for 30 min (NMLA: *n* = 9 mice, NMDA: *n* = 10). (**f**) Time course analysis of the biting response (NMLA: *n* = 9 mice, NMDA: *n* = 10). (**g**) Quantification of the total number of biting responses during 60 min (NMLA: *n* = 9 mice, NMDA: *n* = 10). (**h**) Time course analysis of social contacts (NMLA: *n* = 9 mice, NMDA: *n* = 10). (**i**) Percentage of social contacts (total number of occurrences divided by total number of observations) during 10 min (NMLA: *n* = 9 mice, NMDA: *n* = 10). (**j**) Raster plots showing the effects of the cMPOA lesion on social contacts. (**k**) Quantification (number of occurrences per min) of behaviors displayed during social reunion over 30 min (NMLA: *n* = 9 mice, NMDA: *n* = 10). (**l**) Time course analysis of social contact (NMLA: *n* = 7 mice, NMDA: *n* = 7). (**m**) Quantification of the total social contact occurrences during 8 h (NMLA: *n* = 7 mice, NMDA: *n* = 7). (**n**) Raster plots showing the effects of cMPOA lesions on contacts. The biting responses and social contact were blocked by cMPOA lesions. Time bins, 15 s. Asterisks in (**f**), (**h**), (**i**), and (**k**) indicate significant differences between two groups after Student's unpaired *t*-test (*$p < 0.05$). Asterisks in (**e**), (**l**), and (**m**) indicate significant differences between two groups after Welch's unpaired *t*-test (*$p < 0.05$, ***$p < 0.001$). Graphs show mean ± SEM. See Supplementary Data for exact *p* values and details of statistical analyses.

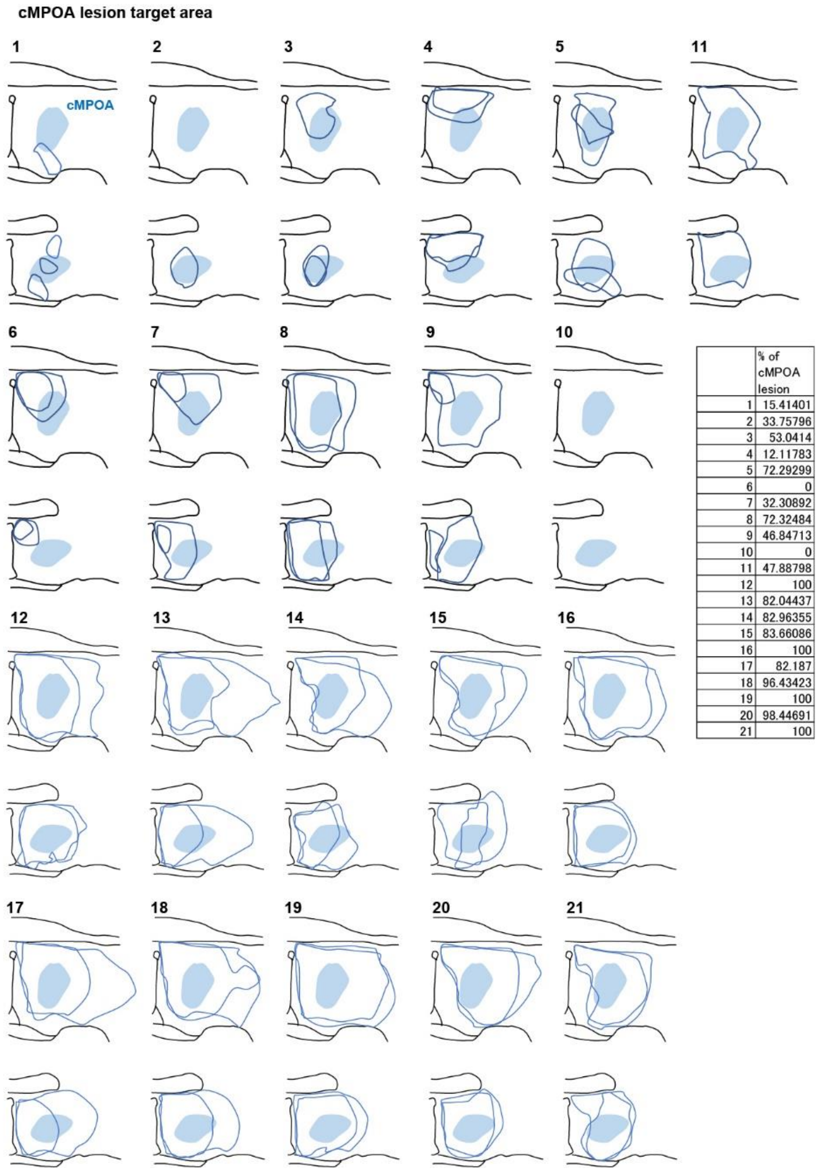

	% of cMPOA lesion
1	15.41401
2	33.75796
3	53.0414
4	12.11783
5	72.29299
6	0
7	32.30892
8	72.32484
9	46.84713
10	0
11	47.88798
12	100
13	82.04437
14	82.96355
15	83.66086
16	100
17	82.187
18	96.43423
19	100
20	98.44691
21	100

Supplementary Figure 6 cMPOA lesion target areas.

Schematic coronal sections showing the cMPOA (blue) and the N-methyl-D-aspartic acid (NMDA)-induced lesion site in the MPOA.

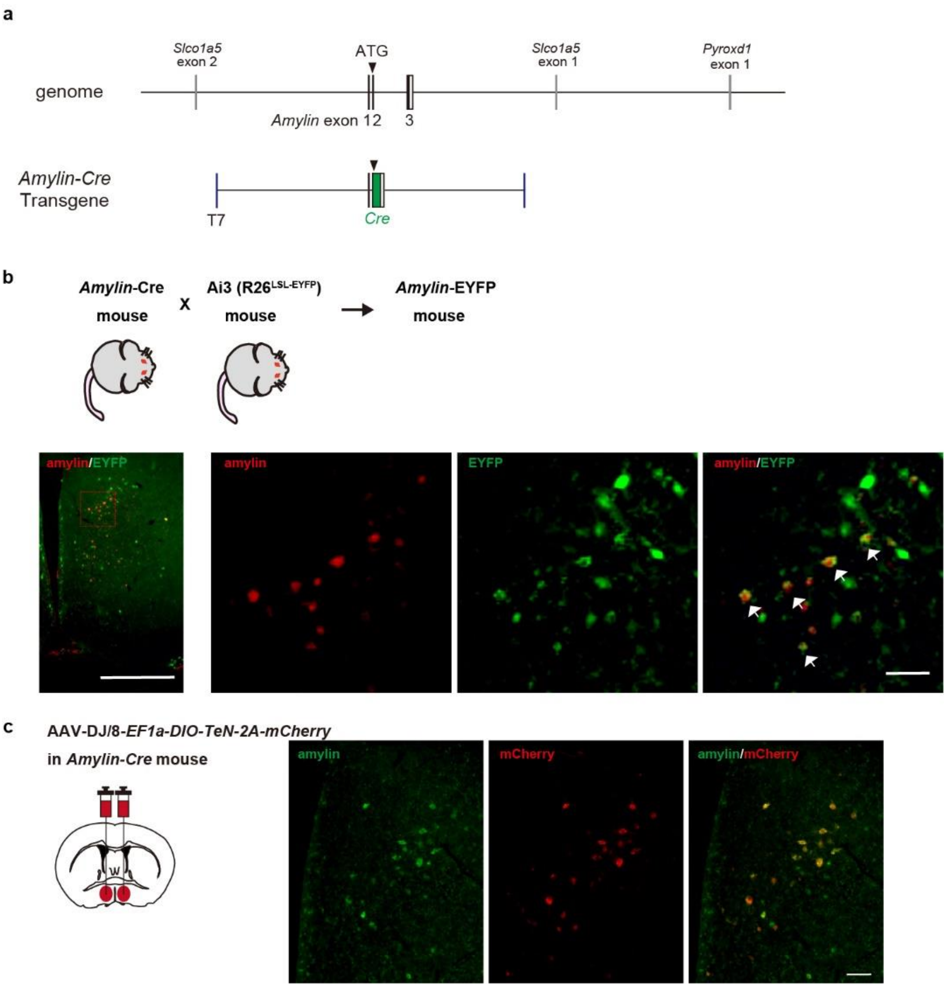

Supplementary Figure 7 Generation of *Amylin-Cre* transgenic mouse line.
(**a**) Structure of *Amylin* gene and *Amylin-Cre* transgenic construct. The Cre sequence was inserted in-frame into the second to third exon. (**b**) Diagram showing the strategy for generating the *Amylin-EYFP* mouse. *Amylin-Cre* mouse was crossed with an Ai3 mouse. Distribution of amylin and EYFP in the MPOA of an *Amylin-EYFP* female mouse. The sections were stained by immunohistochemistry using an anti-amylin antibody. Arrowheads indicate amylin and EYFP double-labeled cells. Scale bars, 500 μm (left) and 50 μm (right). (**c**) AAV-DJ/8-*EF1a-DIO-TeN-2A-mCherry* donated by the McHugh's laboratory was bilaterally injected in the cMPOA of *Amylin-Cre* mice. The sections were stained by immunohistochemistry using an anti-amylin antibody. Scale bar, 50 μm.

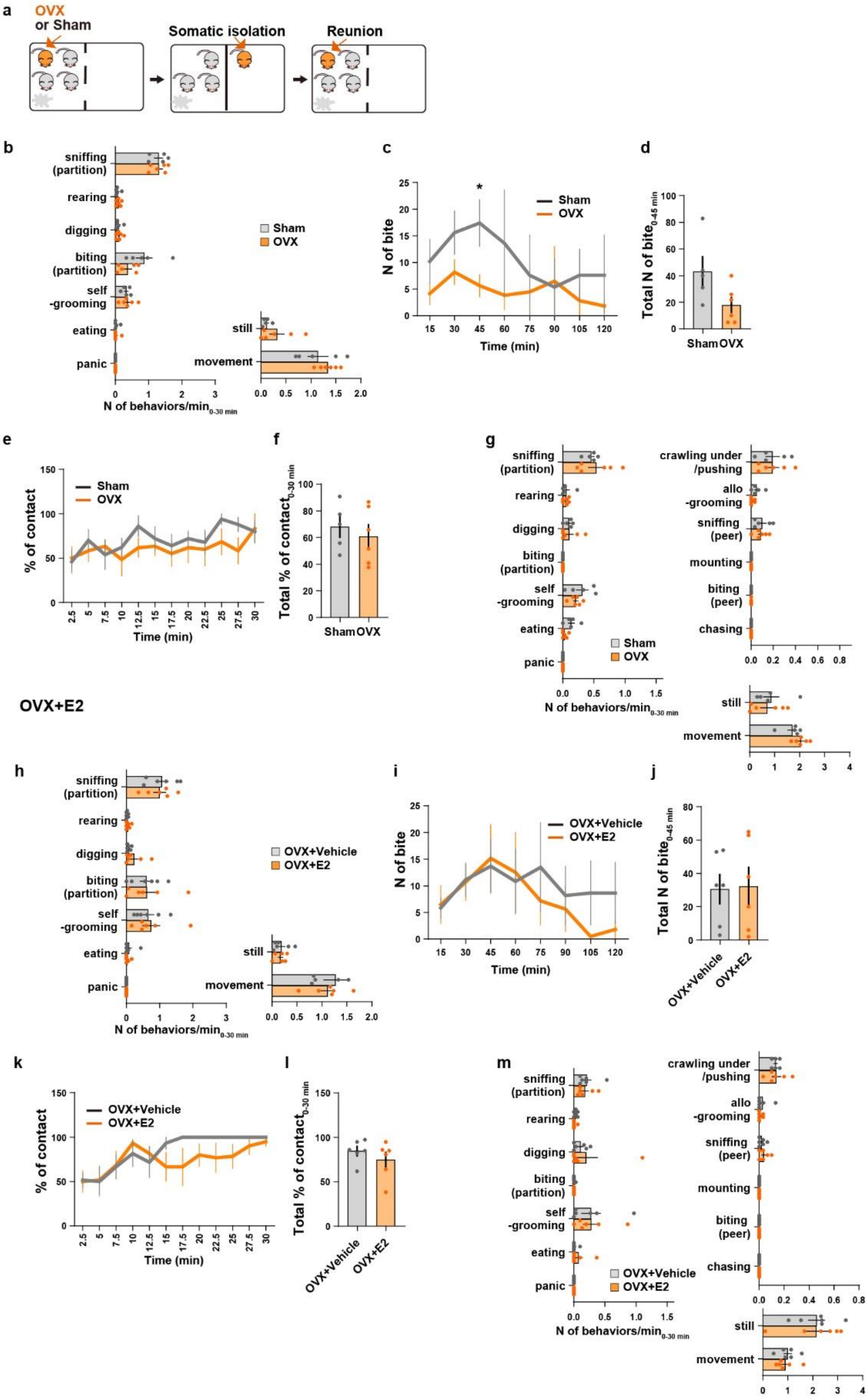

Supplementary Figure 8 Ovariectomy disrupts contact-seeking behaviors induced

by social isolation.

(**a**) Behavioral experiment procedure. Effect of ovariectomy (OVX) (**b–g**), or estradiol supplement in OVX mice (**h–m**) on behaviors (Sham: n = 5 mice, OVX, OVX+Vehicle, and OVX+E2: n = 6). (**b, h**) Quantification (number N of occurrences per min) of behaviors displayed during somatic isolation for 30 min. (**c,i**) Time course analysis of biting responses. (**d, j**) Quantification of the total number of biting responses during 45 min. (**e, k**) Time course analysis of social contacts. (**f, l**) Percentage of social contacts (total number of occurrences divided by total number of observations) during 30 min. (**g, m**) Quantification (number of occurrences per min) of various behaviors displayed during social reunion for a 30 min period. Time bins: 15 sec. Asterisks in (**c**) indicate significant differences between sham and OVX groups (Mann-Whitney U test, two-sided, $*p < 0.05$). Graphs show mean ± SEM. See Supplementary Data for exact p values and details of statistical analyses.

Supplementary Discussion

Definition of the terms "Social Behavior," "Social," "Sociable," and "Sociality."

The more knowledgeable and rigorous the scholar is, like Edward O. Wilson [1] or Robert Hinde [2], the more hesitant s/he is to clearly define social behavior. In the natural language, "social behaviors" are behaviors involving two or more individuals of the same species and do not include behaviors such as sleep, urination, or self-grooming. The ethologists, however, have found ambiguous examples, ranging from bacterial conjugation, swarms of lake flies, timed egg and sperm release by sea urchins, to urination performed by an isolated animal but with communicative roles. For practical purposes, here we use the definition of "social behavior" proposed previously [3], as "any action directed by an individual towards a member of its own species." It follows that, "It (social behavior) includes both competitive behavior, such as fighting, threat and submission, and co-operative (or affiliative in the chapter 4 of the same book) interactions such as parental care and mating. All mammals show social behavior, however infrequent their contact with other members of their own species, because internal fertilization necessitates mating, and lactation involves an intimate relationship between mother and unweaned young." Similar definitions and statements have been made by others [4, 5]. Not surprisingly, this rough definition will not classify marginal cases unambiguously.

While we use this wide definition of "social behavior", we avoid using an adjective "social" to describe an animal species that performs at least some social behaviors, as it is not very meaningful to call all the mammalian species as "social". Indeed, by far the majority of mammals are so-called solitary [1, 3]; that is, the adult individuals stay alone for most of their lives, except for the short period of mating and mother-infant interactions before weaning. The adjective "social" is often used to mean "non-solitary", when used as an adjective of an animal species or taxon (see the chapter 23 of [1]), and we follow this tradition. For the purpose of this paper, here we also use a more specific term "sociable", as in the chapter 5 of [3], to mean the tendency for adult animals to stay together for a certain period of time in an affiliative manner, but not merely for reproductive purposes or for agonistic/competitive purposes. By this definition of the term "sociable", we include social interactions with at least some affiliative social behaviors, and exclude the prolonged period of territorial marking or competitive fighting

among individuals. Again, this rough definition will not clearly classify ambiguous cases, such as a play interaction that transforms into a real fight. “Prosocial”, as used in [4], is a tempting alternative for the same purpose, but it is also used as antonym of “antisocial” in human psychology, and in this case, limited to altruistic behaviors.

A "sociable" trait links to the concept of "sociality", that is simply defined as cooperative group living (the chapter 1 of [5]). Based on the framework originally derived from primate studies [6], the social system (society) is a set of conspecific animals interacting regularly and is characterized by three elements: social organization (group composition), social structure (how group members interact), and mating system. Care should be taken when using the terms "social" or "sociable" for an animal species, therefore, because there are at least three independent aspects for mammalian sociality; mother-infant, mating, and non-reproductive group living. For example, species A is monogamous and a breeding pair lives in isolation, while species B is polygamous, live in a large group and nurse communally. It is not possible to determine which species is more social. Moreover, most social traits in mammals are very labile and flexible phylogenetically or within populations, and within individuals [1 7].

The term "social homeostasis" has been coined and widely used for insect societies [8, 9], and also is applied recently in a rather different context for individual mice [10]. We prefer not to use this term for the amylin-Calcr functions in sensing and seeking for social contacts in mice; firstly it is still unclear whether the two phenomena, insect social homeostasis and mouse tendency to maintain social contacts, share the underlying neural mechanisms; secondly, the term "social allostasis" could be more useful for the extensive flexibility of mouse sociality as described above and in the main text.

References

1. Wilson, E.O. *Sociobiology: the new synthesis* (Belknap Press, 1975).

2. Hinde, R.A. *Biological bases of human social behaviour* (McGraw-Hill, 1974).

3. Poole, T.B. *Social behaviour in mammals* (Blackie ;
Distributed in the USA by Chapman and Hall, Glasgow
New York, 1985).

4. Numan, M. *Neurobiology of Social Behavior: Toward an understanding of prosocial and antisocial brain* (Elsevier, London, 2015).

5. Rubenstein, D.R. & Abbot, P. *Comparative social evolution* (Cambridge University Press, Cambridge, United Kingdom, 2017).

6. Kappeler, P.M. & van Schaik, C.P. Evolution of primate social systems. *International Journal of Primatology* **23**, 707-740 (2002).

7. Kappeler, P.M., Barrett, L., Blumstein, D.T. & Clutton-Brock, T.H. Constraints and flexibility in mammalian social behaviour: introduction and synthesis. *Philos Trans R Soc Lond B Biol Sci* **368**, 20120337 (2013).

8. Emerson, A.E. Regenerate Behavior and Social Homeostasis of Termites. *Ecology* **37**, 248-258 (1956).

9. Holldobler, B. & Wilson, E.O. *The ant* (Harvard University Press, Cambridge, 1990).

10. Lee, C.R., Chen, A. & Tye, K.M. The neural circuitry of social homeostasis: Consequences of acute versus chronic social isolation. *Cell* **184**, 1500-1516 (2021).
