# Supplementary material for: Amylin-Calcitonin receptor signaling in the medial preoptic area mediates affiliative social behaviors in female mice
Source: Nat Commun. 2022 Feb 8;13:709. doi: 10.1038/s41467-022-28131-z (PMC8825811; doi:10.1038/s41467-022-28131-z)
Supplement: Supplementary file 3 — Description of Additional Supplementary Files [file 41467_2022_28131_MOESM3_ESM.pdf]

Description of Additional Supplementary Files

File Name: Supplementary Data 1

Description: Statistical analyses.
