# Supplementary material for: Amylin-Calcitonin receptor signaling in the medial preoptic area mediates affiliative social behaviors in female mice
Source: Nat Commun. 2022 Feb 8;13:709. doi: 10.1038/s41467-022-28131-z (PMC8825811; doi:10.1038/s41467-022-28131-z)
Supplement: Supplementary file 5 — reporting summary [file 41467_2022_28131_MOESM5_ESM.pdf]

nature research

Corresponding author(s): Kansai Fukumitsu and Kumi O. Kuroda

Last updated by author(s): Dec 23, 2021

Reporting Summary

Nature Research wishes to improve the reproducibility of the work that we publish. This form provides structure for consistency and transparency in reporting. For further information on Nature Research policies, see our Editorial Policies and the Editorial Policy Checklist.

Statistics

For all statistical analyses, confirm that the following items are present in the figure legend, table legend, main text, or Methods section.

n/a	Confirmed	
☐	☒	The exact sample size (n) for each experimental group/condition, given as a discrete number and unit of measurement
☐	☒	A statement on whether measurements were taken from distinct samples or whether the same sample was measured repeatedly
☐	☒	The statistical test(s) used AND whether they are one- or two-sided<br>*Only common tests should be described solely by name; describe more complex techniques in the Methods section.*
☐	☒	A description of all covariates tested
☐	☒	A description of any assumptions or corrections, such as tests of normality and adjustment for multiple comparisons
☐	☒	A full description of the statistical parameters including central tendency (e.g. means) or other basic estimates (e.g. regression coefficient) AND variation (e.g. standard deviation) or associated estimates of uncertainty (e.g. confidence intervals)
☐	☒	For null hypothesis testing, the test statistic (e.g. F, t, r) with confidence intervals, effect sizes, degrees of freedom and P value noted<br>*Give P values as exact values whenever suitable.*
☒	☐	For Bayesian analysis, information on the choice of priors and Markov chain Monte Carlo settings
☒	☐	For hierarchical and complex designs, identification of the appropriate level for tests and full reporting of outcomes
☐	☒	Estimates of effect sizes (e.g. Cohen's d, Pearson's r), indicating how they were calculated

Our web collection on statistics for biologists contains articles on many of the points above.

Software and code

Policy information about availability of computer code

Data collection	The web-based software siDirect (version 2.0) was used to design shRNAs. For microscopy, image data was taken by NanoZooomer Digital Pathology (Hamamatsu Photonics), BZ 9000 All in one Fluorescence Microscope (Keyence) and confocal microscopy FV1000 (Olympus).<br>For mouse behavioral analysis, video was recorded using video camera (iVIS HF R52, Canon), infrared camera (400-CAM035-2, Sanwa Direct) and interval camera (recolo, KINGGIM) , and when necessary, 15-sec, 1-min or 5-min interval still images were collected by Batch Video to Image Extractor V0.1.7.<br>Locomotor activity and core temperature were recorded using nanotag (Kissei Comtec Co).
Data analysis	Statistics were performed using Excel 2013 (Microsoft) , Prism 8 (GraphPad) and software R (R Development Core Team, 2019).<br>Histochemical data was analyzed by ImageJ (version 1.52a, NIH).<br>Continuous video data of mouse behavior was analyzed by using CAPTIV-L2100 (TEA).

For manuscripts utilizing custom algorithms or software that are central to the research but not yet described in published literature, software must be made available to editors and reviewers. We strongly encourage code deposition in a community repository (e.g. GitHub). See the Nature Research guidelines for submitting code & software for further information.

Data

Policy information about availability of data

All manuscripts must include a data availability statement. This statement should provide the following information, where applicable:

- Accession codes, unique identifiers, or web links for publicly available datasets
- A list of figures that have associated raw data
- A description of any restrictions on data availability

The authors declare that all data supporting the findings of this study are available within the paper and its supplementary information files. Source data are

provided with this paper.

Field-specific reporting

Please select the one below that is the best fit for your research. If you are not sure, read the appropriate sections before making your selection.

☒ Life sciences ☐ Behavioural & social sciences ☐ Ecological, evolutionary & environmental sciences

For a reference copy of the document with all sections, see nature.com/documents/nr-reporting-summary-flat.pdf

Life sciences study design

All studies must disclose on these points even when the disclosure is negative.

Sample size	No statistical methods were used to determine sample sizes. Sample sizes for each experiment were determined based on our previous studies (EMBO J 34., 2015; Cell Rep 35., 2021) and were described in the figure legends.
Data exclusions	All the data were included, except for those with unsuccessful sampling, such as damaged brain tissue for histology or technical errors of video recording.
Replication	All the data were from at least two independent subsets of experiments, found to show a similar trend, and thus joined to the full sample. All attempts at replication were successful. The biological replicate number corresponds to the number of mice. Technical replicates were not performed in this study. Individual data is presented for transparency.
Randomization	Randomly selected mice from a litter were assigned to each experimental group to avoid selection and litter biases. The sequential and spatial order of the experimental procedure, including the behavioral testing, was randomized among experimental groups.
Blinding	The behavioral data were quantified in an experimenter-blind setting. c-Fos expressing neurons (Fig. 4) were automatically counted by Image J. Other histological analyses were not blinded, due to the high complexity of these tasks.

Reporting for specific materials, systems and methods

We require information from authors about some types of materials, experimental systems and methods used in many studies. Here, indicate whether each material, system or method listed is relevant to your study. If you are not sure if a list item applies to your research, read the appropriate section before selecting a response.

Materials & experimental systems

n/a	Involved in the study	
☐	☒	Antibodies
☐	☒	Eukaryotic cell lines
☒	☐	Palaeontology and archaeology
☐	☒	Animals and other organisms
☒	☐	Human research participants
☒	☐	Clinical data
☒	☐	Dual use research of concern

Methods

n/a	Involved in the study	
☒	☐	ChIP-seq
☒	☐	Flow cytometry
☒	☐	MRI-based neuroimaging

Antibodies

Antibodies used	anti-c-Fos (1:5,000, sc-52, Santa Cruz Biotechnology), anti-Calcr (1:4,000, PAb188/10, Welcome receptor antibodies), anti-amylin (1:20,000, cat#H-017-11, Phoenix Pharmaceuticals), anti-NeuN (1:5000, cat#MAB377, Merck Millipore), anti-ERa (1:20,000, cat#06-935, Merck Millipore), anti-Neurophysin I (NPI) antibody (1:2,000, sc-7810, Santa Cruz Biotechnology), anti-GM130 (1:1000, cat#610822, BD Biosciences), anti-DIG (1:10000, cat#11333089001, Roche Diagnostics), anti-mCherry(1:5,000, ab167453, Abcam), anti-GFP antibody (1:5,000, cat#598, MBL),<br>biotin-conjugated anti-rabbit (BA-1100), mouse (BA-2000) and goat(BA-9500) secondary antibody (1:2000,Vector Laboratories), Alexa 488- or Alexa 568-conjugated anti-rabbit, anti-mouse, or anti-goat IgG (1:1000, cat#ab150077, ab150113, A21206, ab175471, ab150129, ab175474, Invitrogen or Abcam), biotin-Streptavidin-Alexa Fluor 568 (1:1000, cat#S11226, Invitrogen)
Validation	The antibodies used in this study are validated by the manufacturers as shown in the following websites and by our previous studies (EMBO J 34., 2015; Cell Rep 35., 2021). Quality of anti-Calcr or amylin was especially validated by ISH in adult female mice (C57BL/6J and BALB/c).<br>anti-c-Fos:https://www.scbt.com/p/c-fos-antibody-4?productCanUrl=c-fos-antibody-4&_requestid=12133601<br>anti-Calcr: http://www.welcomereceptor.com/antibodies-of-choice/<br>anti-amylin:https://www.phoenixpeptide.com/products/view/Antibodies/H-017-11<br>anti-NeuN: https://www.merckmillipore.com/JP/en/product/Anti-NeuN-Antibody-clone-A60,MM_NF-MAB377

anti-ERa:https://www.merckmillipore.com/JP/en/product/Anti-Estrogen-Receptor-Antibody,MM_NF-06-935?ReferrerURL=https%3A%2F%2Fwww.google.com%2F
anti-Neurophysin I (NPI): https://www.scbt.com/p/neurophysin-i-antibody-m-15?requestFrom=search
anti-GM130:https://www.bdbiosciences.com/en-us/products/reagents/microscopy-imaging-reagents/immunofluorescence-reagents/purified-mouse-anti-gm130.610822
anti-DIG: https://www.sigmaaldrich.com/JP/en/product/roche/11333089001
anti-mCherry: https://www.abcam.com/mcherry-antibody-ab167453.html
anti-GFP:https://ruo.mbl.co.jp/bio/e/dtl/A/?pcd=598
biotin-conjugated anti-rabbit secondary antibody: https://vectorlabs.com/biotinylated-horse-anti-rabbit-igg-antibody.html
biotin-conjugated anti-mouse secondary antibody:https://vectorlabs.com/biotinylated-horse-anti-mouse-igg-antibody.html
biotin-conjugated anti-goat secondary antibody:https://vectorlabs.com/biotinylated-horse-anti-goat-igg-antibody.html
Alexa 488--conjugated anti-rabbit IgG: https://www.abcam.com/goat-rabbit-igg-hl-alexa-fluor-488-ab150077.html
Alexa 488--conjugated anti-mouse IgG: https://www.abcam.com/goat-mouse-igg-hl-alexa-fluor-488-ab150113.html
Alexa 568--conjugated anti-rabbit IgG: https://www.abcam.com/goat-rabbit-igg-hl-alexa-fluor-568-ab175471.html
Alexa 488--conjugated anti-goat IgG: https://www.abcam.com/donkey-goat-igg-hl-alexa-fluor-488-ab150129.html
Alexa 568--conjugated anti-goat IgG: https://www.abcam.com/donkey-goat-igg-hl-alexa-fluor-568-ab175474.html
Alexa 488--conjugated anti-rabbit IgG:https://www.thermofisher.com/antibody/product/A-21206.html?ef_id=Cj0KCQiAnuGNBhCPARIsACbnLzoKIEAzGdfdd1B_viBCRaGMG_0d6BGYsvC4WZmq8N5_5EqcQvSNc9MaAuKiEALw_wcB:G:s&s_kwcid=AL!3652!3!516608152221!!!g!!&cid=bid_pca_aus_r01_co_cp1359_pjt0000_bid00000_0se_gaw_dy_pur_con&gclid=Cj0KCQiAnuGNBhCPARIsACbnLzoKIEAzGdfdd1B_viBCRaGMG_0d6BGYsvC4WZmq8N5_5EqcQvSNc9MaAuKiEALw_wcB
biotin-Streptavidin-Alexa Fluor 568: https://www.thermofisher.com/order/catalog/product/S11226?SID=srch-hj-S11226

Eukaryotic cell lines

Policy information about cell lines

Cell line source(s)	293FT cell line (Invitrogen, R70007)
Authentication	Low passage (no more than 15) 293FT cells were used for AAV production. None of the cell lines were authenticated.
Mycoplasma contamination	The cell line was not tested for mycoplasma contamination.
Commonly misidentified lines (See ICLAC register)	No commonly misidentified cell lines were used.

Animals and other organisms

Policy information about studies involving animals; ARRIVE guidelines recommended for reporting animal research

Laboratory animals	Amylin-Cre mice (C57BL/6J and BALB/c), Amylin-KO mice (BALB/c), Amylin-EYFP mice (C57BL/6J) and WT mice (C57BL/6J and BALB/c) of both sexes were used for this study . Cg-Gt(ROSA)26Sortm3(CAG-EYFP)Hze/J mice (C57BL/6J) of both sexes were used to produce Amyllin-EYFP mice. C57BL/6J and BALB/c mice raised in our breeding colony were housed in groups of four or five after weaning at 4 weeks. All mice were more than 12 weeks at the start of experiments.
Wild animals	No wild animals were used in the study.
Field-collected samples	No field collected samples were used in the study.
Ethics oversight	All experiments involving mice were performed in agreement with guidelines of the Animal Experiment Committee of RIKEN.

Note that full information on the approval of the study protocol must also be provided in the manuscript.
